# Early Life Supraphysiological Levels of Oxygen Exposure Permanently Impairs Hippocampal Mitochondrial Function

**DOI:** 10.1038/s41598-019-49532-z

**Published:** 2019-09-16

**Authors:** Manimaran Ramani, Kiara Miller, Jamelle Brown, Ranjit Kumar, Jegen Kadasamy, Lori McMahon, Scott Ballinger, Namasivayam Ambalavanan

**Affiliations:** 1 Departments of Pediatrics, University of Alabama at Birmingham, Birmingham, AL 35233 USA; 2 Departments of Bioinformatics, University of Alabama at Birmingham, Birmingham, AL 35233 USA; 3 Departments of cell, Developmental, and Integrative Biology, University of Alabama at Birmingham, Birmingham, AL 35233 USA; 4 Departments of Neurobiology, University of Alabama at Birmingham, Birmingham, AL 35233 USA; 5 Departments of Pathology, University of Alabama at Birmingham, Birmingham, AL 35233 USA

**Keywords:** Energy metabolism, Cellular neuroscience

## Abstract

Preterm infants requiring prolonged oxygen therapy often develop cognitive dysfunction in later life. Previously, we reported that 14-week-old young adult mice exposed to hyperoxia as newborns had spatial and learning deficits and hippocampal shrinkage. We hypothesized that the underlying mechanism was the induction of hippocampal mitochondrial dysfunction by neonatal hyperoxia. C57BL/6J mouse pups were exposed to 85% oxygen or room air from P2–P14. Hippocampal proteomic analysis was performed in young adult mice (14 weeks). Mitochondrial bioenergetics were measured in neonatal (P14) and young adult mice. We found that hyperoxia exposure reduced mitochondrial ATP-linked oxygen consumption and increased state 4 respiration linked proton leak in both neonatal and young adult mice while complex I function was decreased at P14 but increased in young adult mice. Proteomic analysis revealed that hyperoxia exposure decreased complex I NDUFB8 and NDUFB11 and complex IV 7B subunits, but increased complex III subunit 9 in young adult mice. In conclusion, neonatal hyperoxia permanently impairs hippocampal mitochondrial function and alters complex I function. These hippocampal mitochondrial changes may account for cognitive deficits seen in children and adolescents born preterm and may potentially be a contributing mechanism in other oxidative stress associated brain disorders.

## Introduction

Many extremely preterm infants often require prolonged periods of supraphysiological oxygen (hyperoxia) exposure for their survival. In addition, even preterm infants not receiving supplemental oxygen are exposed to a relatively hyperoxemic environment compared to the hypoxemic normal intrauterine environment (PO_2_ 25–35 mm Hg) during a critical developmental period for many organ systems. Preterm infants who require prolonged periods of oxygen supplementation are at higher risk of morbidities such as retinopathy of prematurity^1,2^ and chronic lung disease like bronchopulmonary dysplasia (BPD)^3,4^, probably as a consequence of chronic oxidative stress (OS). Children with BPD frequently exhibit deficits in executive function and cognition even in the absence of apparent brain injuries such as intraventricular hemorrhage and periventricular leukomalacia^5–8^. Although direct effects of OS and lung injury-induced systemic inflammation on the developing brain have been considered as possible etiologies, the exact mechanism(s) by which children with BPD develop cognitive dysfunction despite no apparent brain injury is not known.

Although the long-term detrimental effects of early hyperoxia exposure on lung development and function have been studied in depth, little is known about long-term effect of early hyperoxia exposure on brain development and function. Previously, we have shown that in C57BL/6J mice, hyperoxia (85% oxygen [O_2_]) exposure during the neonatal period (P2–14) (neonatal hyperoxia) leads to spatial memory and learning deficits, increased exploratory behavior, and shrinkage of area CA1 of the hippocampus when assessed at young adult age (14 weeks)^9^. Recently, our proteomic analysis of hippocampal homogenates from neonatal mice (P14) exposed to hyperoxia from P2–14 indicated impairments in hippocampal protein synthesis and translation and predicted mitochondrial dysfunction^10^. Hyperoxic exposure can cause cell death^11^ and impair cell survival^12^ in the developing brain. Chronic OS due to O_2_ supplementation may negatively affect neuronal mitochondrial function and lead to neurodegenerative disorders^13^.

Area CA1, a region of the hippocampus crucial for the acquisition of long-term memory^14–16^, is highly vulnerable to OS^17^. Mitochondria isolated from CA1 neurons have been shown to generate more reactive oxygen species (ROS) than any other regions of the hippocampus^18^. Adequate mitochondrial function is essential for mechanisms required for learning and memory^19^ in the hippocampus. While mitochondrial dysfunction is associated with the pathogenesis of several neurodegenerative diseases in adults^20^, the impact of early-life mitochondrial dysfunction on long-term brain development and function is yet to be determined.

In this study, we hypothesized that prolonged hyperoxia exposure during the critical developmental period would permanently alter hippocampal mitochondrial function. Our objective was to determine the long-term changes in hippocampal mitochondrial respiratory complex protein expression and bioenergetic function in neonatal mice (P14) and young adult mice (14 weeks) exposed to hyperoxia from P2–P14.

## Results

### Targeted and global proteomics

#### Long-term effect of neonatal hyperoxia exposure on hippocampal mitochondrial complex I, II, and III protein expressions in young adult mice

Young adult mice exposed to hyperoxia as neonates had reduced amounts of complex I NADH Dehydrogenase [Ubiquinone] 1 Beta Subcomplex 8 (NDUFB8), and complex I NADH Dehydrogenase [Ubiquinone] 1 Beta Subcomplex 11 (NDUFB11) subunit proteins (Table 1). The levels of other detected complex I subunits and of complex II subunits were comparable between the groups (Table 1). Complex III cytochrome b-c1 complex subunit 9 was increased in the hyperoxia-exposed group compared to air-exposed group (Table 1).

**Table 1:** Long-term Effect of Neonatal Hyperoxia Exposure on Hippocampal Mitochondrial Complex I, II and III Proteins in Young Adult Mice (n = 5 in Air group, 6 in Hyperoxia group).

Molecule (Symbol)	Protein Log Fold Change in Hyperoxia (vs. Air)	P value for protein change
**Complex I Protein Subunits**		
NADH Dehydrogenase [Ubiquinone] 1 Alpha Subcomplex		
Subunit 2 (NDUFA2)	−0.27	0.81
Subunit 5 (NDUFA5)	+0.65	0.38
Subunit 6 (NDUFA6)	−0.18	0.90
Subunit 7 (NDUFA7)	+1.34	0.23
Subunit 8 (NDUFA8)	+2.19	0.08
Subunit 9 (NDUFA9)	+0.82	0.51
Subunit 10 (NDUFA10)	−0.54	0.65
Subunit 12 (NDUFA12)	+1.36	0.36
Subunit 13 (NDUFA13)	+1.29	0.26
Assembly Factor 3 (NDUFAF3)	−0.90	0.58
Assembly Factor 4 (NDUFAF4)	+0.26	0.85
Assembly Factor 5 (NDUFAF5)	+0.09	0.94
NADH Dehydrogenase [Ubiquinone] 1 Beta Subcomplex		
Subunit 3 (NDUFB3)	+0.02	0.98
Subunit 4 (NDUFB4)	−0.35	0.79
Subunit 5 (NDUFB5)	+0.42	0.78
Subunit 7 (NDUFB7)	+1.10	0.46
**Subunit 8 (NDUFB8)**	−**2.65**	**0.04**
Subunit 10 (NDUFB10)	+0.37	0.63
**Subunit 11, (NDUFB11)**	−**2.37**	**0.034**
NADH Dehydrogenase [Ubiquinone] 1		
Subunit C2 (NDUFC2)	+0.07	0.95
NADH Dehydrogenase [Ubiquinone]		
Flavoprotein 1 (NDUFV1)	+1.94	0.17
Flavoprotein 2 (NDUFV2)	+0.54	0.19
**Complex II Protein Subunits**		
Succinate dehydrogenase		
Cytochrome b560 subunit (SDHC)	+0.89	0.53
Iron-sulfur subunit (SDHB)	+0.82	0.49
Flavoprotein subunit (SDHA)	−0.14	0.64
**Complex III Protein Subunits**		
Cytochrome B5 Type B (CYB5B)	−0.38	0.78
Cytochrome b-c1 complex subunit 9 (UQCR10)	+**4.44**	**0.0005**

#### Long-term effect of neonatal hyperoxia exposure on hippocampal mitochondrial complex IV and V protein expressions in young adult mice

Young adult mice exposed to hyperoxia as neonates had less cytochrome C oxidase subunit 7B (COX7B) protein compared to air-exposed groups (Table 2). The amounts of other detected complex IV and V subunits were similar between the groups (Table 2).

**Table 2:** Long-term Effect of Neonatal Hyperoxia Exposure on Hippocampal Mitochondrial Complex IV and V Proteins in Young Adult Mice (n = 5 in Air group, 6 in Hyperoxia group).

Molecule (Symbol)	Protein Log Fold Change in Hyperoxia (vs. Air)	P Value for Protein Change
**Complex IV Protein Subunits**		
Cytochrome C Oxidase		
Subunit 2 (COX2)	+0.67	0.06
Subunit 4 Isoform 1 (COX41)	+0.42	0.59
Subunit 5A (COX5A)	−1.42	0.33
Subunit 5B (COX5B)	+0.15	0.78
Subunit 6A1 (CX6A1)	+3.32	0.08
Subunit 6B1 (CX6B1)	+0.55	0.34
Subunit 6C (COX6C)	+0.19	0.87
Subunit 7A2 (CX7A2)	+0.96	0.51
**Subunit 7B (COX7B)**	−**2.09**	**0.04**
Subunit 7C (COX7C)	+0.39	0.39
Assembly Factor 7 (COA7)	+1.76	0.18
Subunit NDUFA4 (NDUA4)	+1.17	0.06
Translational Activator 1 (TACO1)	−0.74	0.58
**Complex V Protein Subunits**		
ATP Synthase		
Protein 8 (ATP8)	+0.66	0.25
Subunit Alpha (ATPA)	+0.16	0.34
Subunit Beta (ATPB)	+0.02	0.93
Subunit Delta (ATPD)	−0.34	0.78
Subunit Gamma (ATPG)	+0.44	0.48
Subunit F (ATPK)	−1.82	0.30
Subunit O (ATPO)	+0.47	0.23
Subunit D (ATP5H)	+0.73	0.06
Subunit G (ATP5L)	−0.33	0.85
Subunit E (ATP5I)	+0.89	0.11
Subunit Epsilon (ATP5E)	−1.35	0.36
Subunit S (ATP5S)	+0.88	0.37

### Bioinformatic analysis of differentially expressed hippocampal proteins

#### Differentially expressed hippocampal proteins in young adult mice following neonatal hyperoxia exposure

**Figure 1:**
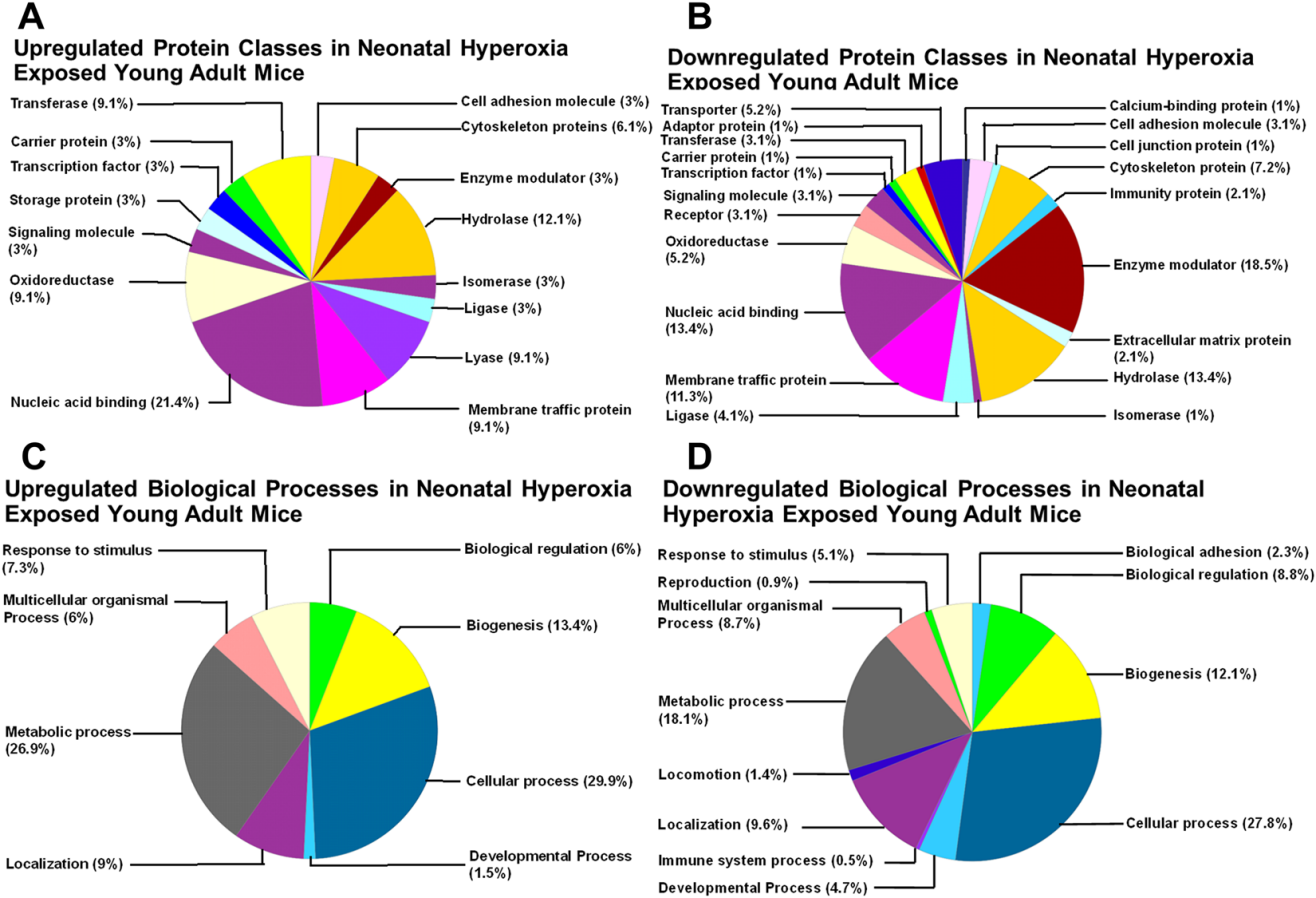
Distribution of hippocampal proteins in young adult mice exposed to neonatal hyperoxia. (**A**) Graphical representation of the distribution of upregulated hippocampal proteins by class in the hyperoxia-exposed group. (**B**) Graphical representation of the distribution of downregulated hippocampal proteins by class in the hyperoxia-exposed group. (**C**) Graphical representation of the distribution of upregulated hippocampal proteins by biological processes in the hyperoxia-exposed group. (**D**) Graphical representation of the distribution of downregulated hippocampal proteins by biological processes in the hyperoxia-exposed group.

With a cut-off of ±1.5 fold-change with P-value < 0.05 (by analysis of variance), and a false discovery rate of 5%, we identified a total of 196 hippocampal proteins that were differentially expressed in neonatal hyperoxia-exposed young adult mice compared to air-exposed young adult mice. Of these 196 proteins, 48 proteins were increased, and 148 were decreased following neonatal hyperoxia exposure. The heat map of the differentially expressed hippocampal proteins is shown in Supplemental Fig. S1. The full list of upregulated and downregulated proteins (limited to fold-change 1.5) in young adult mice exposed to neonatal hyperoxia are listed in Supplemental Tables S1 and S2, respectively. The top 10 differentially expressed hippocampal proteins in the hyperoxia-exposed young adult mice are listed in Table 3. The protein classes that are upregulated and downregulated by hyperoxia exposure are shown in Fig. 1A,B, respectively.

**Table 3:** Top 10 Differentially Expressed Hippocampal Proteins in Neonatal Hyperoxia-Exposed Young Adult Mice (n = 5 in Air group, 6 in Hyperoxia group, P = Hyperoxia vs. Air group).

Protein (Symbol)	Log Fold Change in Hyperoxia Group	P value
**Up-regulated Proteins**		
Ras-related protein Rab-8A (RAB8A)	+4.78	0.032
Cytochrome b-c1 complex subunit 9 (UQCR9)	+4.44	0.0005
Regulator complex protein LAMTOR3 (LTOR3)	+4.35	0.02
Mitochondrial Ribosomal Protein L11 (MRPL11)	+4.19	0.003
Myelin proteolipid (PLP)	+4.05	0.003
**Down-regulated Proteins**		
Filamin-C (FLNC)	−5.13	9.97E-07
Vacuolar Protein Sorting-Associated Protein 52 Homolog (VPS52)	−4.74	1.79E-06
Integrin Beta-1 (ITB1)	−4.50	5.77E-07
Glucose-6-Phosphate 1-Dehydrogenase X (G6PD1)	−4.44	0.01
Teneurin-1 (TEN1)	−4.38	0.0001

#### Long-term effect of effect of neonatal hyperoxia exposure on hippocampal biological processes in young adult mice

Differentially expressed hippocampal proteins were predominantly involved in biological processes of cellular process, metabolic process, biogenesis, and protein localization (Fig. 1C,D). Among the upregulated hippocampal proteins, functions of 20 (29.9%) proteins were associated with cellular process, 18 (26.9%) were proteins associated with metabolic process, 9 (13.4%) were proteins associated with the biogenesis process, and 6 (9%) were proteins associated with the localization process (Fig. 1C). Among the downregulated proteins, functions of 62 (27.8%) proteins were associated with the cellular process, 39 (18.1%) were proteins associated with metabolic process, 26 (12.1%) were proteins associated with biogenesis process, and 20 (9.6%) were proteins associated to localization process (Fig. 1D).

#### Top canonical hippocampal pathways regulated by neonatal hyperoxia exposure in young adult mice

The top canonical pathways that were most impacted by neonatal hyperoxia exposure are listed in Table 4. Bioinformatic analysis of differentially expressed hippocampal proteins predicated that mitochondrial function (P = 2.03E-06), oxidative phosphorylation (P = 1.25E-05), GABA receptor signaling (P = 1.26E-04), amyotrophic lateral sclerosis signaling (P = 4.09E-04), and amyloid processing (P = 5.34E-04) were impacted in young adult mice that had neonatal hyperoxia exposure. Fifteen proteins were associated with mitochondrial function, 11 were related to oxidative phosphorylation, 9 were related to GABA receptor signaling, 9 were proteins related to amyotrophic lateral sclerosis signaling, and 6 were proteins related to amyloid processing and were differentially expressed in the hyperoxia-exposed group.

**Table 4:** Top Canonical Pathways Involved in Neonatal Hyperoxia-Exposed Young Adult Mice by Ingenuity Pathway Analysis (Using Proteomics Data, n = 5 in Air group, 6 in Hyperoxia group, P = Hyperoxia vs. Air group).

Name	P-Value	Overlap (percentage; number of proteins differentially expressed/number of proteins in pathway)
Mitochondrial dysfunction	2.03E-06	8.8; 15/171
Oxidative phosphorylation	1.25E-05	10.1; 11/109
GABA receptor signaling	1.26E-04	9.5; 9/95
Amyotrophic lateral sclerosis signaling	4.09E-04	8.1; 9/111
Amyloid processing	5.34E-04	11.8; 6/51

### Mitochondrial studies

#### Effects of neonatal hyperoxia exposure on hippocampal mitochondrial bioenergetics in neonatal mice (P14)

**Figure 2:**
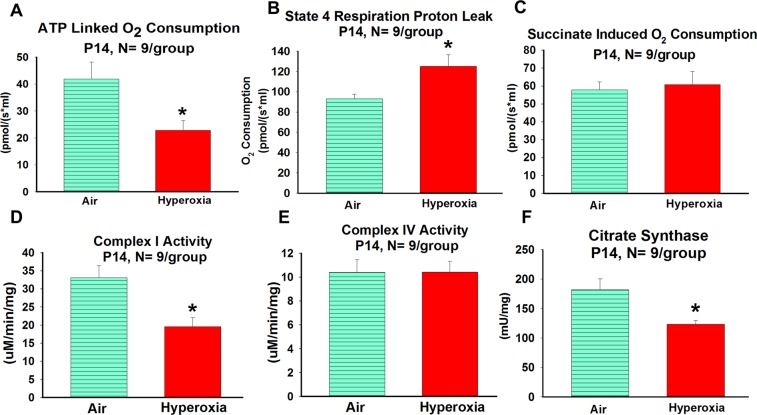
Effects of neonatal hyperoxia exposure on hippocampal mitochondrial bioenergetics in neonatal mice. **A** = ATP linked oxygen consumption, **B** = State 4 respiration proton leak, **C** = Succinate induced oxygen consumption, **D** = Complex I activity measured by assay, **E** = Complex IV activity measured by assay, and **F** = Citrate synthase activity measured by assay. Air-exposed: cyan bars with horizontal stripes and hyperoxia-exposed: solid red bars; means ± SEM; n = 9 in air and 9 in hyperoxia. *p < 0.05 vs. air-exposed mice.

Neonatal hyperoxia (exposure from P2–P14) exposure decreased both pyruvate/malate mitochondrial ATP linked (P = 0.01) and complex I enzyme activity (P = 0.01) at P14 (Fig. 2A,D, respectively). No differences were observed between hyperoxia-exposed and air-exposed controls in ATP linked O_2_ consumption rates that utilized succinate (complex II substrate; P = 0.73) or complex IV activity (P = 0.99) (Fig. 2C,E, respectively), consistent with the hypothesis that the observed differences were related to a complex I defect. Examination of state 4 minus basal O_2_ consumption rates also suggested increased oxygen consumption in the hyperoxia-exposed group (P = 0.03), which could be linked to increased proton leak and/or oxidant generation (Fig. 2B). Hyperoxia-exposed neonatal mice also had reduced citrate synthase activity (P = 0.01) (Fig. 2F), compared to air-exposed neonatal mice.

#### Effects of neonatal hyperoxia exposure on hippocampal mitochondrial bioenergetics in young adult mice (14 weeks)

**Figure 3:**
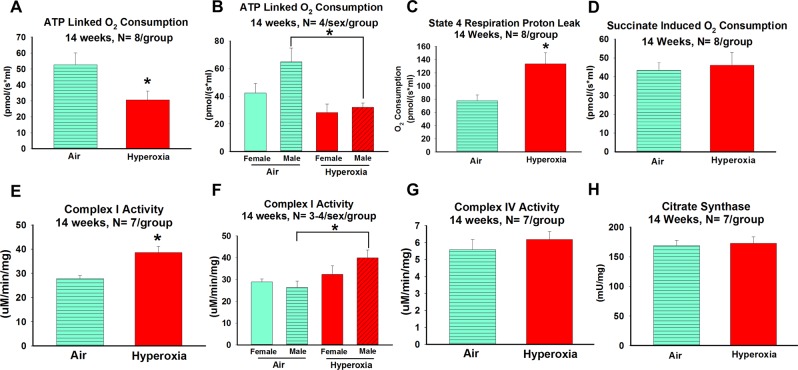
Effects of neonatal hyperoxia exposure on hippocampal mitochondrial bioenergetics in young adult mice. **A** = ATP linked oxygen consumption, **B** = ATP linked oxygen consumption by sex, **C** = State 4 respiration proton leak, **D** = Succinate induced oxygen consumption, **E** = Complex I activity measured by assay, **F** = Complex I activity by Sex, **G** = Complex IV activity measured by assay, and **H** = Citrate synthase activity measured by assay. (**A,C,D,E,G** and **G**); air-exposed: cyan bars with horizontal stripes and hyperoxia-exposed: solid red bars; means ± SEM; n = 7–8 in air and 7–9 in hyperoxia. *p < 0.05 vs. air-exposed mice. (**B,F**); Air-exposed females: solid cyan bars, air-exposed males: cyan bars with horizontal stripes, hyperoxia-exposed females: solid red bar, and hyperoxia-exposed males, red bars with angled stripes; means ± SEM; n = 4/sex/group. *p < 0.05 = air-exposed mice vs. hyperoxia-exposed males by One Way ANOVA.

Similar to the observations in neonates exposed to hyperoxia, adult mice (14 weeks old) that underwent neonatal hyperoxia exposure from P2–P14 had decreased mitochondrial ATP linked O_2_ consumption in the presence of complex I substrates (pyruvate/malate) (P = 0.01) (Fig. 3A) whereas in the presence of succinate (P = 0.74), no differences were observed relative to air-exposed controls (Fig. 3D). Similarly, no differences were observed in complex IV activity (P = 0.45) between exposed and unexposed control groups (Fig. 3G). Also, similar to the hyperoxia-exposed newborn mice, oligomycin induced state 4 O_2_ consumption rates minus basal O_2_ consumption rates were increased (P = 0.05) (Fig. 3C), consistent with increased proton leak and/or oxidant generation. However, unlike in neonates, complex I activity (P = 0.002) was significantly increased in the 14-week old mice exposed to hyperoxia as neonates (Fig. 3E). Subgroup analysis by sex also showed that young adult male mice exposed to hyperoxia as neonates had decreased ATP linked O_2_ consumption (One Way ANOVA, mean difference 36.7, P = 0.01) (Fig. 3B) and increased complex I activity (One Way ANOVA, mean difference 13.66, P = 0.03) (Fig. 3F) compared to air-exposed young adult male mice. The difference in citrate synthase activity (P = 0.78) seen in neonatal mice exposed to hyperoxia were no longer observed when assessed as young adults (Fig. 3H).

#### Effects of neonatal hyperoxia exposure on hippocampal mitochondrial copy number in neonatal (P14) and young adult mice (14 weeks)

**Figure 4:**
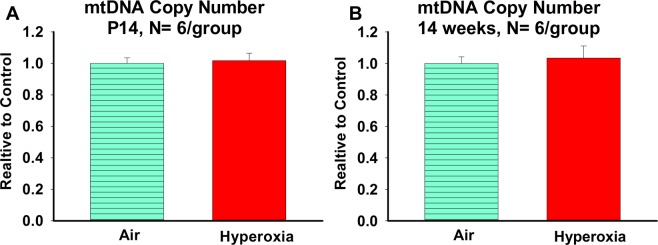
Effects of neonatal hyperoxia exposure on hippocampal mitochondrial DNA copy number in neonatal and young adult mice. **A** = Mitochondrial copy number relative to air exposed controls in neonatal (P14) mice and **B** = Mitochondrial copy number relative to air exposed controls in young adult (P14) mice. Air-exposed: cyan bars with horizontal stripes and hyperoxia-exposed: solid red bars; means ± SEM; n = 6 in air and 6 in hyperoxia. *p < 0.05 vs. air-exposed mice.

No difference in the mitochondrial copy number was observed among the air- and hyperoxia-exposed neonatal mice (Fig. 4A). Similarly, no difference in the mitochondrial copy number was observed among air- and hyperoxia-exposed young adult mice (Fig. 4B).

## Discussion

This is the first preclinical study to demonstrate the long-term adverse effect of early life hyperoxia on hippocampal mitochondrial function and mitochondrial respiratory chain protein expression. We discovered that hyperoxia exposure during a critical developmental period permanently impairs hippocampal mitochondrial function, alters the expression of specific respiratory chain subunits for complexes I and III, and impairs complex I activity in the hippocampus. As spatial memory deficits and other cognitive problems in the mouse model of bronchopulmonary dysplasia (BPD) correspond to the cognitive deficits seen in adolescents with BPD, these new observations suggest that permanent hippocampal mitochondrial dysfunction induced by early life oxygen exposure as a contributor to the pathophysiology of BPD associated cognitive dysfunction.

This study has several strengths. We have used unbiased proteomic analysis of whole hippocampal tissue using highly sensitive mass spectrometric methods. Rather than being limited to only mitochondrial proteins, our study also evaluated the long-term impact of early life oxygen exposure on all hippocampal proteins and used sophisticated bioinformatics analysis to define long-term changes in the hippocampal signaling pathways. This study also evaluated mitochondrial bioenergetics induced by hyperoxia exposure during the critical developmental period (P14) and young adult age (14 weeks, the age at which we observed cognitive dysfunction in our previous study). In addition to citrate synthase assay, a surrogate measure of mitochondrial content, we also measured mitochondrial copy number, an alternative measure for mitochondrial content.

There are also a few limitations to this study. The proteomics and the mitochondrial bioenergetic studies were performed on the whole hippocampus instead of specific hippocampal subfields which are known to play different roles in memory and learning. Furthermore, since proteomic and bioenergetic studies were done from the whole hippocampus, it is not possible to determine whether these early life oxygen-induced changes in hippocampal proteins and mitochondrial function were predominantly derived from neurons or glial cells or a combination of both. In addition, proteomic methods often require large sample sizes, as there is often much variation from one sample to another, and even large differences may not end up being statistically significant. In this study, we focused on proteomics and mitochondrial bioenergetics only from the hippocampal homogenates, and not from other regions of the brain such as cerebellum, amygdala, corpus callosum, and white matter tracts which might also have impacted by hyperoxia exposur^21–23^. Even though hippocampal complexes I and IV activities were measured, complex III and complex V activities were not measured due to technical difficulties and the size of the hippocampus.

Though lung and brain development in newborn mouse pups corresponds to 24–28 weeks of gestation in human preterm infants, the highly efficient redox and gas exchange system of the C57BL6 mice^24^ requires supraphysiological concentrations (85% O_2_) and a longer duration (P2–14) of oxygen exposure to induce human BPD-like lung pathology^9,25^. Our model, while not an exact simulation of the human preterm infant in the neonatal intensive care unit, reproduces both structurally the hippocampal shrinkage and functionally the associated memory deficits^26^ seen in adolescents and young adults with BPD.

The hippocampus, a region of the brain that plays a vital role in consolidating short memory into long-term memory^14–16^, is highly vulnerable to oxidative stress^17^. Oxygen exposure causes neuronal cell death in developing brain^11,12^, and prolonged oxidative stress impairs neuronal mitochondrial function^13^. Neurons in the hippocampus are critically dependent on their mitochondrial function for the strengthening of synapses, a cellular response responsible for the formation and maintenance of long-term memory^27–29^. In neurons, mitochondria generate about 90% of the ATP by oxidative phosphorylation. In oxidative phosphorylation, oxygen is the terminal electron acceptor of the mitochondrial electron transport chain (ETC). ETC transfers electrons from high energy metabolites through a series of electron acceptors (carriers) to drive the generation of ATP from ADP^30^. The redox state of the respiratory chain is governed by the trans-membrane proton gradient and the membrane potential^31^. The redox energy used for ATP generation also leads to the production ROS^32^. Excessive ROS production following hyperoxia exposure can potentially overwhelm antioxidant defense mechanisms and leads to mitochondrial damage^33,34^ and cellular death^35^.

Our mitochondrial functional assessments show that early life hyperoxia exposure not only reduces ATP linked oxygen consumption in the hippocampus in the neonatal period (P14) but also in the young adult (14 weeks). In addition, our study also shows a persistent increase in the rate of oxygen consumption at state 4 respiration, (a surrogate measure of proton leak) in young adults exposed to hyperoxia as neonates, and suggests uncoupling between substrate oxidation and ATP synthesis. Alterations in mitochondrial coupling can alter ROS production^36–38^ and ATP synthesis^39^. Though the amount of ATP produced by the hippocampal tissue was not measured in this study, the decrease in ATP linked oxygen consumption and increase in state 4 proton leak both at P14 and 14 weeks suggest that early life oxygen exposure permanently impairs mitochondrial efficiency in the generation of ATP. The neonatal hyperoxia-induced hippocampal mitochondrial dysfunction measured through bioenergetic studies in young adult mice is consistent with the mitochondrial dysfunction predicted through proteomic analysis.

Complex I (NADH: ubiquinone oxidoreductase), the first and largest enzyme in the ETC, has been consistently shown to be vulnerable to oxidative stress-mediated dysfunction^40^. It is also thought to be the main site of ROS production^41,42^, and its impairment leads to an increase in ROS production^43^. Decreased complex I activity seen in hyperoxia-exposed neonatal mice suggests that oxygen exposure either directly or indirectly impairs complex I function. At 14 weeks, the targeted hippocampal proteomic analysis determined decreases in complex I NDUFB8 and NDUFB11 subunits in neonatal hyperoxia-exposed mice, inner membrane subunits that are located in the membrane arm of complex I together along with proton pumping subunits. While neither of these subunits is thought to be directly involved in catalysis, decreased levels of NDUFB8 associated with AD in rodent model^44^. However, mitochondrial bioenergetic studies indicated an increase in complex I activity at 14 weeks. The persistent decreased ATP linked O_2_ consumption and increased state 4 proton leak at 14 weeks despite the increase in complex I activity in the young adult mice exposed to hyperoxia suggest persistent mitochondrial dysfunction and inadequate compensation by the later increase in complex I activity following hyperoxia-induced decreases in the newborn. Neonatal hyperoxia did not affect complex IV activity either at P14 or 14 weeks suggesting that either the complex IV enzyme is not as highly vulnerable to oxidative stress as complex I or it is well adapted to oxidative stress-induced injury. Though changes in hyperoxia-induced complex III and V activity are possible, comparable succinate-induced oxygen consumption between hyperoxia and air-exposed neonatal and young adult mice indicate that dysfunction in oxygen consumption noted with hyperoxia exposure is mainly induced by alterations in complex I function.

Mitochondrial metabolism and signaling pathways that regulate cell death are sexually dimorphic^45^. Compared to the female, the male hippocampus has a lower level of endogenous antioxidant defense systems^46^ and produces more ROS^47^. We determined that hyperoxia-exposed young males had reduced ATP linked O_2_ consumption and increased complex I activity compared to hyperoxia-exposed young females. These observations are clinically important because prematurity associated neurodevelopmental outcomes^48^ and neurodevelopmental disorders (e.g., Autism)^49^ preferentially affects male sex. Citrate synthase, a surrogate marker for mitochondrial volume^50^, was reduced by hyperoxia in neonates (P14) and normalized in young adults (14 weeks). However, when we independently verified the citrate synthase (mitochondrial content) results with mitochondrial copy number by qPCR, we did not observe any differences in mitochondrial copy number among the groups both either in neonatal or young adult mice. This suggests that even though early life hyperoxia exposure permanently impairs hippocampal mitochondrial function, it may not have significant short-term or long-term impact on hippocampal mitochondrial biogenesis.

In addition, hyperoxia-induced changes in the expression of Ras-related protein Rab-8A (involved in vesicular trafficking and neurotransmitter), Teneurin-1 (increases hippocampal dendritic arborization and spine density) and their roles in hyperoxia-induced cognitive dysfunction need further investigation. Also, reduction in hippocampal glucose-6-phosphate 1-dehydrogenase X (G6PD1) level in hyperoxia-exposed young adult mice suggests that early life hyperoxia exposure permanently impairs cytosolic oxidative phosphorylation, a process that is critical for the NADPH production. Since adequate NADPH level is essential for cellular oxidative stress regulation^51^, it is possible that impaired hippocampal oxidative phosphorylation in hyperoxia-exposed young adult mice could have also impaired its ability to defend against oxidative stress even under normal ambient air conditions.

Our data also indicate that aberrant GABAergic signaling^52^ and amyloid processing are associated with cognitive deficits, and these pathways have been linked to neurodegenerative conditions^53^. Additional studies are needed to evaluate the contribution of these canonical pathways to impaired memory and hippocampal dysfunction induced by oxidative stress and to define how they interact with mitochondrial dysfunction. Since mitochondrial biogenesis is not impacted by early hyperoxia, we speculate that early oxidative stress possibly alterted complex I protein structure leading to an increase in mitochondrial ROS production which in turn may contribute to oxidative damage to mitochondrial DNA, altered mitophagy, and mitochondrial structure leading to long-term changes in complex I function and overall mitochondrial function (Supplemental Fig. S2). It is also possible that neonatal hyperoxia-induced phenotype might have originated not only from the initial insult (direct oxidative stress) to the complex I and other ETC enzymes but also due to the signaling pathways such as mitochondrial UQCR9, MRPL11 RAB8A, G6PD, and Teneurin-1 that are poorly compensated at a later point.

## Conclusion

This study demonstrated that supraphysiological oxygen exposure during a critical period in neonatal development has a permanent negative impact on hippocampal mitochondria. The pathophysiology of neonatal hyperoxia-induced permanent mitochondrial dysfunction is complex. Future studies designed to quantitate mitochondrial DNA damage, ATP, and ROS levels are needed to determine the mechanisms by which early hippocampal complex I dysfunction induces permanent complex I dysfunction and the development of spatial memory deficits.

## Materials and Methods

All protocols were approved by the UAB Institutional Animal Care and Use Committee (IACUC) and were consistent with the PHS Policy on Humane Care and Use of Laboratory Animals (Office of Laboratory Animal Welfare, Aug 2002) and the Guide for the Care and Use of Laboratory Animals (National Research Council, National Academy Press, 1996).

### Animal model

**Figure 5:**
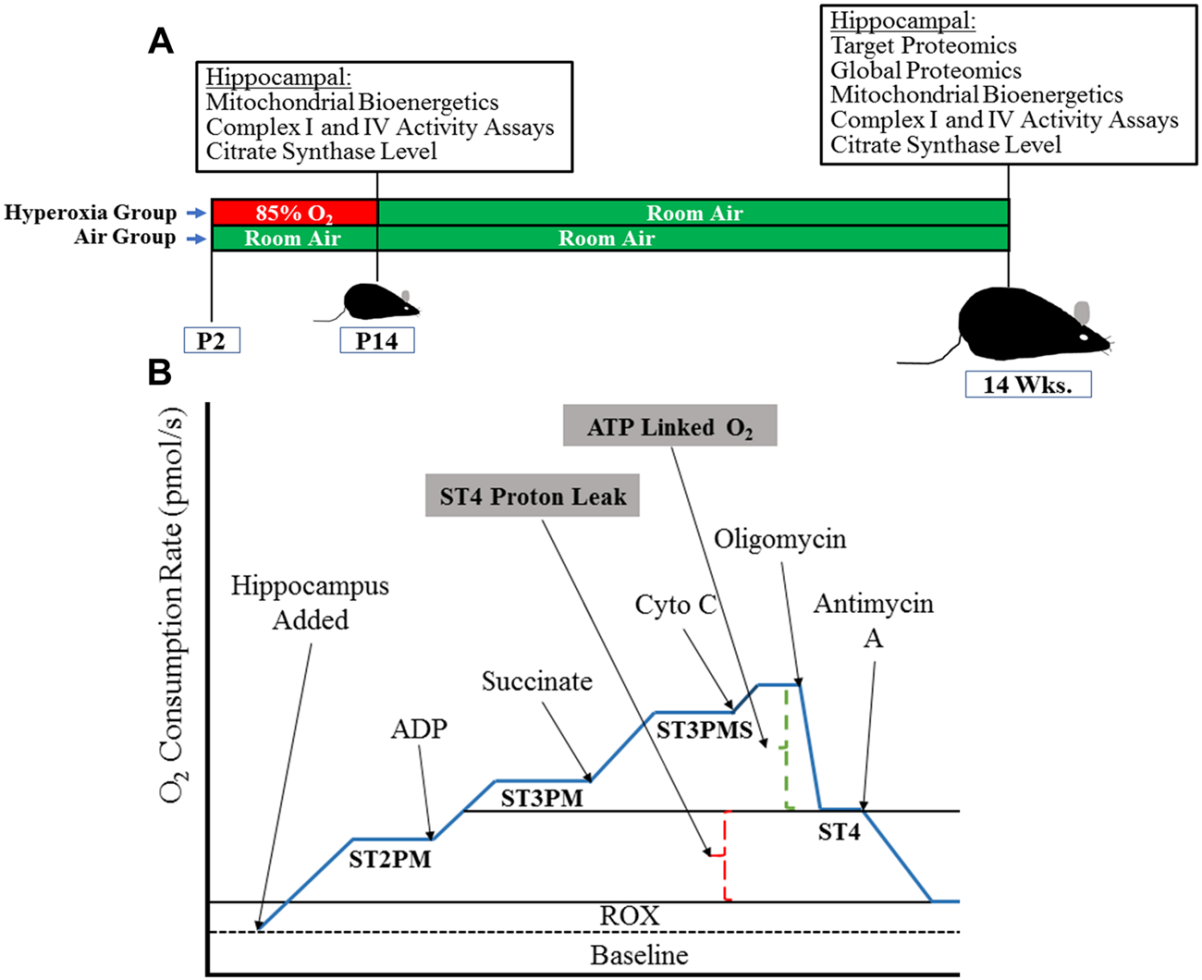
Schematics of the animal model and mitochondrial respiratory protocol. (**A**) Represents schematic of hyperoxia exposure from P2–14 and experimental studies done at P14 and 14 weeks. (**B**) Represents schematic of the mitochondrial respiratory protocol used in the high-resolution respirometry with sequentially added substrates and the calculations to assess the mitochondrial bioenergetic function using whole hippocampal tissue. ST2PM = State 2 respiration with pyruvate and malate, ST3PM = State 3 respiration with pyruvate and malate, ADP = Adenosine diphosphate, ST3PMS = State 3 respiration with pyruvate, malate and succinate, ST4 = State 4 respiration following oligomycin, ATP Linked O_2_ = Adenosine triphosphate linked O_2_ consumption, ST4 Proton Leak = State 4 respiration proton leak, ROX = Residual O_2_ consumption, and Baseline = Baseline O_2_ consumption.

C57BL/6J dams and their pups of both sexes were exposed to either normobaric hyperoxia (85% O_2_, N = 6) or normobaric 21% O_2_ ambient air (Air, N = 6) from the second postnatal day (P2) until postnatal day 14 (P14), returned to room air, and maintained on standard rodent diet and light/dark cycling in microisolator cages until 14 weeks of age (Fig. 5A)^25^. An additional set of mice were exposed to either 85% O_2_ (Hyperoxia, N = 6) or 21% O_2_ (Air, N = 6) and sacrificed at P14 (Fig. 5A).

At 14 weeks, hippocampal proteins were analyzed using unbiased proteomic profiling using mass spectrometry. Initially, the targeted protein analysis was performed for hippocampal mitochondrial respiratory complex proteins. Subsequently, bioinformatics analysis was conducted on all other differentially expressed hippocampal proteins between hyperoxia and air-exposed groups. At P14 and 14 weeks of age, hippocampal tissues were analyzed for mitochondrial bioenergetic functions, complex I, IV, citrate synthase activity, and mitochondrial copy number.

#### Mass spectrometry

At 14 weeks, following cervical dislocation, the whole brain was harvested from the mice, and hippocampi were removed in a sterile manner^10^. Then, Tissue was homogenized using Qiagen tissue lyser (Qiagen, MD, USA) in T-PER + Halt protease inhibitors + PMSF solution, and protein assay was performed using BCA protein assay kit (Thermo Fisher Scientific, MA, USA)^54^. The mass spectrometric analysis of hippocampal proteins was performed as previously described^10^.

#### Proteomics data assessment

Differentially expressed proteins (fold change ± 1.5 fold and p < 0.05) were identified using T-test and further analyzed. As previously done^10^, functional analysis was performed using PANTHER (Protein ANalysis THrough Evolutionary Relationships)^55^ and Ingenuity Pathway Analysis (QIAGEN Inc. MD, USA). Heat maps were generated using pheatmap package V.1.0.7 in R program.

### Mitochondrial bioenergetic studies (high-resolution respiratory)

Whole hippocampus (right) was harvested and placed in ice cold artificial cerebrospinal fluid that contained glucose, BSA, EGTA, pyruvate, and mitochondrial respiration buffer, as previously described^56^. Briefly, hippocampal tissue was permeabilized with saponin (5 mg/mL, 30 minutes) and high-resolution respirometry performed using a two-channel respirometer (Oroboros Oxygraph-2k with DatLab software; Oroboros, Innsbruck, Austria). Reactions were conducted at 37 °C in a 2 ml chamber containing air-saturated mitochondrial respiration buffer (MiR03) under continuous stirring.

As illustrated in Fig. 4B, O_2_ consumption rates were measured in the presence of substrates (5 mM malate, 15 mM pyruvate, 2.5 mM ADP,10 mM succinate), and inhibitors (0.5 μM oligomycin, 5um antimycin A) to assess state 2 (substrate alone), state 3 (substrate + ADP) and oligomycin induced state 4 respiration rates. Non-mitochondrial oxygen consumption was determined in the presence of antimycin A. Adenosine triphosphate (ATP) linked O_2_ consumption rate was determined by State 3 (substrates + ADP) - State 4 (oligomycin) = ATP linked rate. Non-ATP linked O_2_ consumption rate was determined by State 4 (oligomycin) – non-mitochondrial oxygen consumption (antimycin A). Potential differences in O_2_ consumption rates based upon substrate utilization at complex I or II were assessed using pyruvate/malate or succinate, respectively, in the presence of ADP.

### Complex I, IV and citrate synthase activity assays

Complex I, IV, and citrate synthase activities were measured from hippocampal (left) homogenates as previously described^57–59^; complex I activities were measured from freshly extracted tissues.

### Mitochondrial DNA copy number (qPCR)

Mitochondrial DNA copy number was determined by QPCR as previously described^60^. Briefly, DNA was extracted from hippocampus homogenates from neonatal (P14) and young adult (14 weeks) mice exposed to room air or hypoxia from P2–14 using a Qiagen DNA® Mini Kit (Qiagen). DNA was quantified via fluorescence using Quant-iT™ PicoGreen™ dsDNA Assay Kit (Invitrogen). 15 ng of DNA from each sample was used for quantitative PCR (qPCR). PCR products underwent electrophoresis for 2 hours at 90 volts on 10% polyacrylamide gels. Gels were stained with ethidium bromide for 45 minutes and imaged on an Amersham^TM^ Imager 600 (GE Healthcare). All samples were loaded in duplicate and mitochondrial DNA copy number was quantified by measuring band intensities for each age group relative to age-matched room air-exposed controls using ImageQuant (GE Healthcare).

### Statistical analysis

Results were expressed as means ± SE. Multiple comparisons testing (Student-Newman-Keuls) was performed if statistical significance (*p* < 0.05) was noted by ANOVA.

## Supplementary information

Supplementary Figures and Tables
