# Supplementary material for: Early Life Supraphysiological Levels of Oxygen Exposure Permanently Impairs Hippocampal Mitochondrial Function
Source: Sci Rep. 2019 Sep 16;9:13364. doi: 10.1038/s41598-019-49532-z (PMC6746707; doi:10.1038/s41598-019-49532-z)
Supplement: Supplementary file 1 — Supplementary Figures and Tables [file 41598_2019_49532_MOESM1_ESM.doc]

**Title:** **Early Life Supraphysiological Levels of Oxygen Exposure Permanently Impairs Hippocampal Mitochondrial Function**

**Abbreviated title:** Oxygen-Induced Hippocampal Mitochondrial Dysfunction

**Author names and affiliations:**

*Manimaran Ramani MD^1^, Kiara Miller, BS^1^, Jamelle Brown, BS^1^, Ranjit Kumar PhD^2^, Jegen Kadasamy^1^, Lori McMahon PhD^3,4^, Scott Ballinger PhD^5^, and Namasivayam Ambalavanan MD^1,2^

From the Departments of Pediatrics^1^, Bioinformatics^2^, Cell, Developmental, and Integrative Biology^3^, Neurobiology^4^, and Pathology^5^ University of Alabama at Birmingham, Birmingham, AL 35233

**Corresponding author:**

*Manimaran Ramani MD, University of Alabama at Birmingham, 176F Suite 9380,

619 South 20^th^ St., Birmingham, AL 35233 USA. Tel: (205) 934-4680 Fax: (205) 934-3100

Email: [mramani@peds.uab.edu](mailto:mramani@peds.uab.edu)

**Supplemental Fig S1:** Hierarchical clustering of differentially expressed hippocampal proteins in room air vs hyperoxia exposed young adult mice. Dendrogram above the heat map depicts hierarchical clustering of the samples (Room air is shown as red, hyperoxia samples cyan). Cluster distance is based on the average distance between all the pairs of objects in the two clusters. Dendrograms for differentially expressed proteins are shown on the left side of the heat map. In the heat maps, red shows increased expression, and blue indicates decreased expression. Expression value intensities are illustrated by color with a range of -2 to 8 on a log scale.


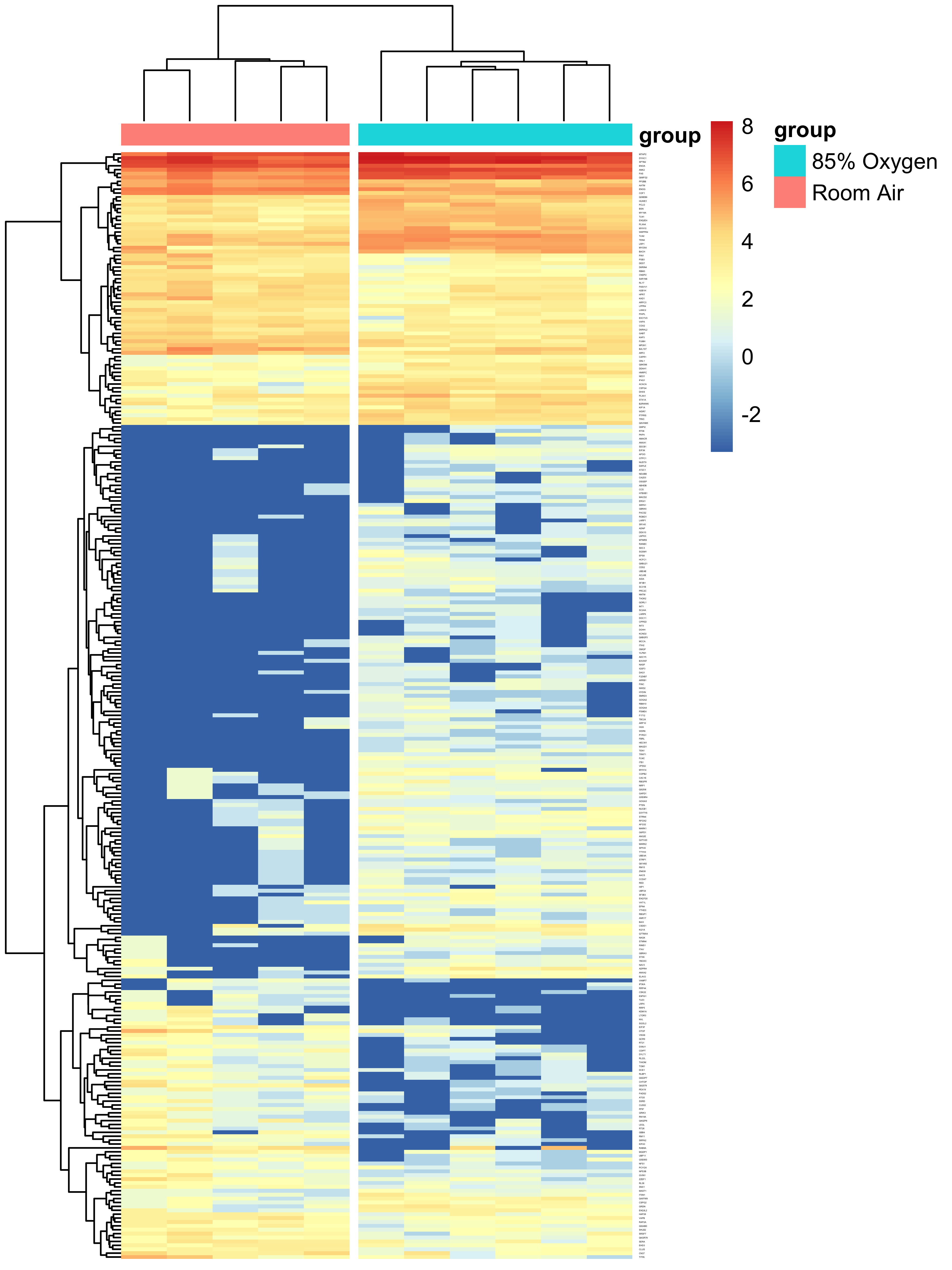


**Supplemental Fig S2**: Cartoon depicting the possible mechanism(s) by which early life oxygen exposure leads to permanent hippocampal mitochondrial dysfunction.


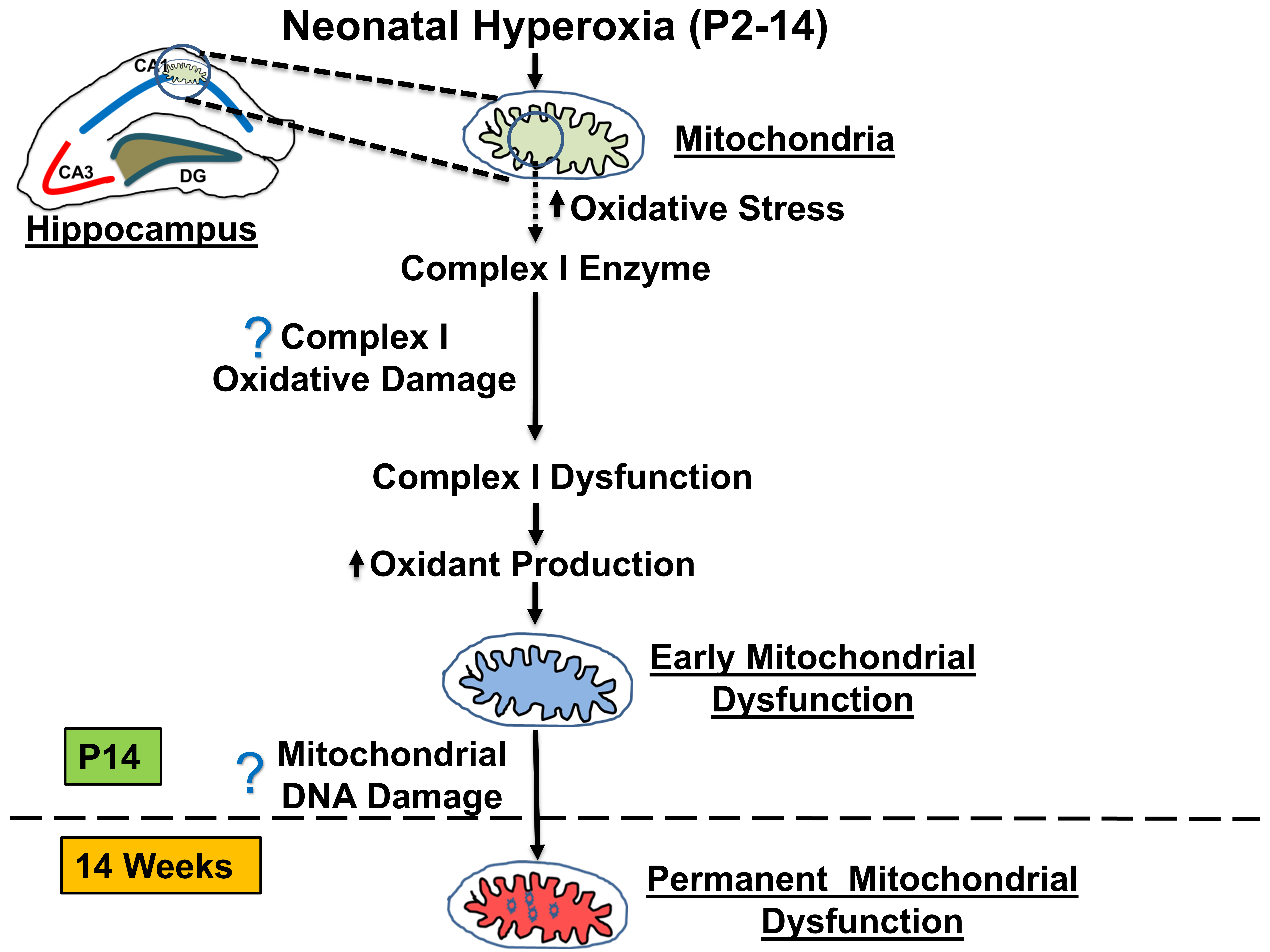


**Supplemental Table S1**: Upregulated Hippocampal Proteins in Young Adult Mice Exposed to Neonatal Hyperoxia (N=6 in Air group, 6 in Hyperoxia group)

| **Molecule (Symbol)** | **Protein**  **Log Fold Change in Hyperoxia (vs. Air)** | **P value for protein change** |
| --- | --- | --- |
| Ras-related protein Rab-8A (RAB8A) | + 4.78 | 0.032 |
| Cytochrome b-c1 complex subunit 9 (UQCR9) | +4.44 | 0.0005 |
| Regulator complex protein LAMTOR3 (LTOR3) | +4.35 | 0.02 |
| 39S ribosomal protein L11, mitochondrial (RM11) | +4.19 | 0.003 |
| Myelin proteolipid (PLP) | +4.05 | 0.003 |
| Protein RRP5 homolog (RRP5) | +3.88 | 0.02 |
| Peptidyl-prolyl cis-trans isomerase F, mitochondrial (PPIF) | +3.87 | 0.003 |
| Otoferlin (OTOF) | +3.74 | 0.01 |
| Vesicle-associated membrane protein 7 (VAMP7) | +3.63 | 0.02 |
| Guanine nucleotide-binding protein subunit beta-4 (GBB4) | +3.61 | 0.04 |
| 28S ribosomal protein S21, mitochondrial (RT21) | +3.60 | 0.01 |
| Transducin-like enhancer protein 3 (TLE3) | +3.54 | 0.02 |
| Translocon-associated protein subunit delta (SSRD) | +3.51 | 0.004 |
| Kinesin-associated protein 3 (KIFA3) | +3.47 | 0.01 |
| Glutamate receptor ionotropic, kainate 3 (GRIK3) | +3.47 | 0.01 |
| SRSF protein kinase 2 (SRPK2) | +3.46 | 0.01 |
| Lysine-specific histone demethylase 1A (KDM1A) | +3.44 | 0.03 |
| GES30 (Q9Z2P7) | +3.42 | 0.01 |
| Low-density lipoprotein receptor-related protein 4 (LRP4) | +3.42 | 0.02 |
| Nuclear valosin-containing protein-like (NVL) | +3.33 | 0.03 |
| Shugoshin-like 2 (SGOL2) | +3.33 | 0.04 |
| Target of Myb protein 1 (TOM1) | +3.20 | 0.01 |
| Casein kinase II subunit alpha (CSNK2A1) | +3.14 | 0.02 |
| Dynein light chain Tctex-type 1 (DYLT1) | +2.98 | 0.02 |
| Inositol-trisphosphate 3-kinase A (IP3KA) | +2.93 | 0.03 |
| Peroxisomal biogenesis factor 19 (PEX19) | +2.92 | 0.01 |
| Galectin-related protein (LEGL) | +2.86 | 0.03 |
| CDP-diacylglycerol--inositol 3-phosphatidyltransferase (CDIPT) | +2.86 | 0.02 |
| E3 ubiquitin-protein ligase RNF19A (RN19A) | +2.84 | 0.02 |
| 60S ribosomal protein L22-like 1 (RL22L) | +2.82 | 0.02 |
| Eukaryotic translation initiation factor 3 subunit F (EIF3F) | +2.79 | 0.04 |
| von Willebrand factor A domain-containing protein 8 (VWA8) | +2.78 | 0.05 |
| Exosome complex exonuclease RRP44 (RRP44) | +2.70 | 0.03 |
| Thioredoxin, mitochondrial (THIOM) | +2.61 | 0.01 |
| Magnesium-dependent phosphatase 1 (MGDP1) | +2.53 | 0.03 |
| Retinaldehyde-binding protein 1 (RLBP1) | +2.53 | 0.03 |
| Armet protein (AMERT) | +2.53 | 0.05 |
| Chromatin target of PRMT1 protein (CHTOP) | +2.43 | 0.05 |
| Ubiquitin carboxyl-terminal hydrolase 11 (UBP11) | +2.43 | 0.01 |
| 28S ribosomal protein S28, mitochondrial (RT28) | +2.41 | 0.03 |
| Glutamate decarboxylase 1 (DCE1) | +2.24 | 0.04 |
| Cytochrome b-5, isoform CRA_a (CYB5B) | +2.10 | 0.05 |
| Fatty acid desaturase 2 (FADS2) | +2.04 | 0.05 |
| Cysteine desulfurase, mitochondrial (NFS1) | +1.98 | 0.0005 |
| Autophagy protein 5 (ATG5) | +1.96 | 0.03 |
| Titin (TITIN) | +1.93 | 0.03 |
| Protein-L-isoaspartate O-methyltransferase (PCMT1) | +1.51 | 0.03 |
| Interferon-induced very large GTPase 1 (GVIN1) | +1.50 | 0.04 |

**Supplemental Table S2:** Downregulated Hippocampal Proteins in Young Adult Mice Exposed to Neonatal Hyperoxia (N=6 in Air group, 6 in Hyperoxia group)

| **Molecule (Symbol)** | **Protein**  **Log Fold Change in Hyperoxia (vs. Air)** | **P value for protein change** |
| --- | --- | --- |
| Filamin-C (FLNC) | -5.13 | 9.97E-07 |
| Vacuolar Protein Sorting-Associated Protein 52 Homolog (VPS52) | -4.74 | 1.79E-06 |
| Integrin Beta-1 (ITB1) | -4.50 | 5.77E-07 |
| Glucose-6-Phosphate 1-Dehydrogenase X (G6PD1) | -4.44 | 0.01 |
| Teneurin-1 (TEN1) | -4.38 | 0.0001 |
| Serine/Threonine-Protein Kinase (MARK1) | -4.17 | 0.01 |
| CCA Trna Nucleotidyltransferase 1(TRNT1) | -4.11 | 9.86E-05 |
| Actin-Like Protein 6B (ACL6B) | -4.07 | 0.001 |
| Cold Shock Domain-Containing Protein E1 (CSDE1) | -4.05 | 0.05 |
| RNA Binding Protein Fox-1 Homolog 2 (RFOX2) | -4.01 | 0.01 |
| Coatomer Subunit Beta 2 (COPB2) | -4.01 | 0.02 |
| E3 Ubiquitin-Protein Ligase HECW1 (HECW1) | -3.97 | 2.16E-05 |
| Nuclear Mitotic Apparatus Protein 1 (NUMA1) | -3.88 | 0.01 |
| Melanoma-Associated Antigen D1 (MAGD1) | -3.87 | 4.92E-05 |
| Glutaminyl-Trna Synthetase, Isoform CRA_A (QARS) | -3.87 | 0.01 |
| U2 Snrnp-Associated SURP Motif-Containing Protein (SR140) | -3.86 | 0.01 |
| Y-Box-Binding Protein 3 (YBOX3) | -3.85 | 0.03 |
| Phosphatidate Cytidylyltransferase 2 (CDS2) | -3.83 | 0.02 |
| CDE1-Binding Protein (CDEBP) | -3.83 | 0.003 |
| AFG3-Like Protein 2 (AFG32) | -3.82 | 0.01 |
| Axin Interactor, Dorsalization-Associated Protein  (AIDA) | -3.79 | 0.01 |
| Ubiquitin Conjugation Factor E4 B (UBE4B) | -3.78 | 0.004 |
| WD Repeat-Containing Protein 6 (WDR6) | -3.78 | 4.21E-05 |
| Acetoacetyl-Coa Synthetase (AACS) | -3.76 | 0.003 |
| Nucleobindin-1 (NUCB1) | -3.76 | 0.01 |
| Endoplasmic Reticulum-Golgi Intermediate Compartment Protein 1 (ERGI1) | -3.75 | 0.01 |
| Fibronectin (FINC) | -3.74 | 0.01 |
| Eukaryotic Translation Initiation Factor 3 Subunit K (EIF3K) | -3.68 | 0.01 |
| ELAV-Like Protein 2 (ELAV2) | -3.68 | 0.04 |
| Striatin-4 (STRN4) | -3.64 | 0.02 |
| Myosin-14 (MYH14) | -3.63 | 0.03 |
| Acidic Leucine-Rich Nuclear Phosphoprotein 32 Family Member E (AN32E) | -3.63 | 0.03 |
| Synaptic Vesicle Membrane Protein VAT-1 Homolog-Like (VAT1L) | -3.62 | 0.04 |
| CTP Synthase 1 (PYRG1) | -3.61 | 0.001 |
| 39S Ribosomal Protein L15, Mitochondrial  (RM15) | -3.60 | 0.004 |
| Protein Tweety Homolog 3 (TTYH3) | -3.60 | 0.003 |
| Coiled-Coil Domain-Containing Protein 47 (CCD47) | -3.53 | 0.003 |
| Beta-Arrestin-1 (ARRB1) | -3.53 | 0.01 |
| Kinesin-Like Protein KIF21A (KI21A) | -3.50 | 0.05 |
| NACHT And WD Repeat Domain-Containing Protein 2 (NWD2) | -3.47 | 0.01 |
| Splicing Factor 3B Subunit 3 (SF3B3) | -3.47 | 0.02 |
| Neuron Navigator 3 (NAV3) | -3.46 | 0.04 |
| Golgin Subfamily A Member 4 (GOGA4) | -3.42 | 0.01 |
| RNA-Binding Protein 10 (RBM10) | -3.42 | 0.004 |
| Oligodendrocyte-Myelin Glycoprotein (OMGP) | -3.37 | 0.01 |
| Rab3 Gtpase-Activating Protein Non-Catalytic Subunit (RBGPR) | -3.36 | 0.03 |
| Golgin Subfamily A Member 2 (GOGA2) | -3.35 | 0.01 |
| Splicing Factor 3B Subunit 1 (SF3B1) | -3.32 | 0.02 |
| Ras Association and Pleckstrin Homology Domains 1 (RAPH1) | -3.31 | 0.01 |
| Brain-Specific Angiogenesis Inhibitor 3 (BAI3) | -3.30 | 0.02 |
| Protein ADP-Ribosylarginine Hydrolase (ADPRH) | -3.29 | 0.04 |
| rRNA 2'-O-Methyltransferase Fibrillarin (FBRL) | -3.28 | 3.61E-05 |
| Ubiquitin Conjugation Factor E4 A (UBE4A) | -3.26 | 0.01 |
| Protein Transport Protein Sec31B (SC31B) | -3.25 | 0.01 |
| Striatin-Interacting Protein 1 (STRP1) | -3.25 | 0.004 |
| Voltage-Dependent R-Type Calcium Channel Subunit Alpha-1E (CAC1E) | -3.24 | 0.03 |
| Apolipoprotein O (APOO) | -3.22 | 0.01 |
| Serine/Threonine-Protein Kinase MARK2 (MARK2) | -3.20 | 0.02 |
| Dedicator Of Cytokinesis Protein 11 (DOC110 | -3.19 | 0.01 |
| Gtpase-Activating Protein And VPS9 Domain-Containing Protein 1 (GAPD1) | -3.17 | 0.03 |
| Regulating Synaptic Membrane Exocytosis Protein 1 (RIMS1) | -3.17 | 0.04 |
| Clathrin Interactor 1 (CLINT1) | -3.16 | 0.02 |
| Hepatocyte Growth Factor-Regulated Tyrosine Kinase Substrate (HGS) | -3.16 | 0.03 |
| Asparagine Synthetase (ASNS) | -3.13 | 0.02 |
| La-Related Protein 6 (LARP6) | -3.12 | 0.01 |
| Elongation Factor Tu GTP Binding Domain Containing 2 (EFTUD2) | -3.11 | 0.04 |
| N-Acetyl-D-Glucosamine Kinase (NAGK) | -3.11 | 0.04 |
| Integrin Alpha-V (ITAV) | -3.07 | 0.03 |
| Syntaxin-6 (STX6) | -3.07 | 0.03 |
| Huntingtin-Interacting Protein 1(HIP1) | -3.05 | 0.04 |
| O-Acetyl-ADP-Ribose Deacetylase MACROD2 (MACD2) | -3.05 | 0.01 |
| Neuropilin-1 (NRP1) | -3.04 | 0.04 |
| Integrator Complex Subunit 1 (INT1) | -3.03 | 0.03 |
| Protein FAM171A2 (F1712) | -3.01 | 0.02 |
| Small G Protein Signaling Modulator 1 (SGSM1) | -2.99 | 0.02 |
| Probable ATP-Dependent RNA Helicase DDX10  (DDX10) | -2.97 | 0.01 |
| Ankyrin Repeat Domain-Containing Protein 17 (ANR17) | -2.97 | 0.02 |
| Proteasome Activator Complex Subunit 4  (PSME4) | -2.95 | 0.03 |
| Stathmin-4 (STMN4) | -2.94 | 0.05 |
| Syntenin-1 (SDCB1) | -2.92 | 0.04 |
| Methylcrotonoyl-Coa Carboxylase Subunit Alpha, Mitochondrial (MCCA) | -2.92 | 0.02 |
| Voltage-Dependent Calcium Channel Subunit Alpha-2/Delta-3 (CA2D3) | -2.92 | 0.03 |
| Plakophilin-4 (PKP4) | -2.91 | 0.03 |
| Gamma-Aminobutyric Acid Receptor Subunit Alpha-5 (GBRA5) | -2.90 | 0.03 |
| Copper Chaperone For Superoxide Dismutase (CCS) | -2.90 | 0.02 |
| Actin-Related Protein 10 (ARP10) | -2.89 | 0.03 |
| TBC1 Domain Family Member 24 (TBC24) | -2.89 | 0.03 |
| OL-Protocadherin Isoform (PCDH10) | -2.89 | 0.05 |
| SAPS Domain Family, Member 3, Isoform CRA_C (SAPS3) | -2.88 | 0.05 |
| Putative GTP Cyclohydrolase 1 Type 2 Nif3l1 (NIF3L1) | -2.88 | 0.04 |
| Proline-Rich and Coiled-Coil-Containing Protein 2C (PRC2C) | -2.87 | 0.05 |
| ADP-Ribose Pyrophosphatase, Mitochondrial (NUDT9) | -2.86 | 0.03 |
| Annexin A2 (ANXA2) | -2.86 | 0.04 |
| Phosphofurin Acidic Cluster Sorting Protein 2 (PACS2) | -2.85 | 0.03 |
| Probable Trna N6-Adenosine Threonylcarbamoyltransferase (OSGEP) | -2.82 | 0.03 |
| Gamma-Aminobutyric Acid Receptor Subunit Alpha-3  (GBRA3) | -2.80 | 0.04 |
| YTH Domain-Containing Family Protein 3 (YTHD3) | -2.80 | 0.02 |
| Splicing Factor 45 (SPF45) | -2.80 | 0.03 |
| Ubiquitin Carboxyl-Terminal Hydrolase 24 (UBP24) | -2.79 | 0.03 |
| Host Cell Factor 1 (HCFC1) | -2.79 | 0.05 |
| Adenylate Cyclase Type 5 (ADCY5) | -2.78 | 0.03 |
| Immunoglobulin Superfamily Member 3 (IGSF3) | -2.77 | 0.03 |
| 28S Ribosomal Protein S6, Mitochondrial (RT06) | -2.76 | 0.03 |
| Zinc Finger Protein 638 (ZN638) | -2.76 | 0.01 |
| Rab Gtpase-Activating Protein 1 (RBGP1) | -2.76 | 0.03 |
| SWI/SNF-Related Matrix-Associated Actin-Dependent Regulator of Chromatin Subfamily D Member 3 (SMRD3) | -2.75 | 0.01 |
| La-Related Protein 1 (LARP1) | -2.75 | 0.03 |
| Ran-Binding Protein 3 (RANB3) | -2.72 | 0.03 |
| Activity-Dependent Neuroprotector Homeobox Protein (ADNP) | -2.71 | 0.004 |
| Phosphatidylinositol 3,4,5-Trisphosphate 3-Phosphatase And Dual-Specificity Protein Phosphatase (PTEN) | -2.71 | 0.05 |
| Alpha/Beta Hydrolase Domain-Containing Protein 11 (ABHDB) | -2.70 | 0.02 |
| Epidermal Growth Factor Receptor Kinase Substrate 8 (EPS8) | -2.70 | 0.03 |
| Myotubularin-Related Protein 9 (MTMR9) | -2.69 | 0.02 |
| Deoxyhypusine Hydroxylase (DOHH) | -2.63 | 0.03 |
| Inter-Alpha-Trypsin Inhibitor Heavy Chain H2 (ITIH2) | -2.60 | 0.03 |
| Serine/Threonine-Protein Kinase TAO2 (TAOK2) | -2.60 | 0.03 |
| Integrator Complex Subunit 3 (INT3) | -2.56 | 0.03 |
| YLP Motif-Containing Protein 1 (YLPM1) | -2.55 | 0.03 |
| Glucosamine-6-Phosphate Isomerase 2 (GNPI2) | -2.54 | 0.03 |
| Casein Kinase I Isoform Alpha (H7BXB1) | -2.53 | 0.03 |
| Dystroglycan (DAG1) | -2.53 | 0.03 |
| Protein Transport Protein Sec24A (SC24A) | -2.53 | 0.03 |
| Syndecan-3 (SDC3) | -2.53 | 0.03 |
| Serine/Threonine-Protein Kinase LMTK3 (LMTK3) | -2.48 | 0.03 |
| Potassium Voltage-Gated Channel Subfamily D Member 2 (KCND2) | -2.47 | 0.03 |
| NAD(P) Transhydrogenase, Mitochondrial (NNTM) | -2.43 | 0.03 |
| Calcium-Transporting Atpase Type 2C Member 1 (AT2C1) | -2.43 | 0.03 |
| Nuclear Autoantigenic Sperm Protein (NASP) | -2.41 | 0.03 |
| NADH Dehydrogenase [Ubiquinone] 1 Beta Subcomplex Subunit 11, Mitochondrial (NDUBB) | -2.37 | 0.03 |
| SRSF Protein Kinase 1 (SRPK1) | -2.37 | 0.03 |
| Quinone Oxidoreductase-Like Protein 1 (QORL1) | -2.34 | 0.03 |
| Golgin Subfamily A Member 3 (GOGA3) | -2.31 | 0.05 |
| Roundabout Homolog 1 (ROBO1) | -2.30 | 0.04 |
| Protein Daple (Daple) | -2.26 | 0.03 |
| Betacstf-64 Variant 3 (B3V097) | -2.25 | 0.04 |
| Annexin A1 (ANXA1) | -2.25 | 0.03 |
| Hydrocephalus-Inducing Protein (HYDIN) | -2.24 | 0.04 |
| Alpha-Methylacyl-Coa Racemase (AMACR) | -2.15 | 0.03 |
| Serine/Threonine-Protein Phosphatase CPPED1 (CPPED) | -2.13 | 0.03 |
| Girdin (GRDN) | -1.76 | 0.02 |
| Acetyl-Coa Carboxylase 1 (ACACA) | -1.75 | 0.04 |
| Chondroitin Sulfate Proteoglycan 4 (CSPG4) | -1.56 | 0.01 |
